# Talent selection in 3 × 3 basketball: role of anthropometrics, maturation, and motor performance

**DOI:** 10.3389/fspor.2024.1459103

**Published:** 2024-09-27

**Authors:** Tim Luca Schmitz, Marie-Therese Fleddermann, Karen Zentgraf

**Affiliations:** Department of Movement Science and Training in Sports, Institute of Sport Sciences, Goethe University Frankfurt, Frankfurt am Main, Germany

**Keywords:** APHV, maturity status, talent development, team sports, talent predictors

## Abstract

**Introduction:**

3 × 3 basketball is becoming more and more professionalized, which is leading to a growing interest in talent development and talent selection. Different studies have demonstrated relevant factors in the talent selection process of 5v5 basketball but not in 3 × 3 basketball. Therefore, this study investigated the main predictors in the talent selection process in 3 × 3 basketball athletes.

**Methods:**

A total of 192 athletes (*M*_age_ = 16.11 ± 0.45 years; *n* = 85 were female) 3 × 3 basketball athletes were assessed for various anthropometric and motor performance variables as well as maturity status. All assessments were carried out during selection camps for the German “under 17” youth national team. Binomial logistic regression was conducted to determine which variables predicted selection (either ‘selected', *n* = 30 female and *n* = 34 male, or ‘non-selected’, *n* = 55 female and *n* = 73 male).

**Results:**

The regression model was statistically significant in female athletes (*χ*² (3) = 26.86, *p* < .001). It explains 37.9% (Nagelkerke's *R*^2^) of the variance in selection status and suggests that the general motor-performance component (*p* < .001) and the anthropometric- and maturation-related component (*p* = .004) seem to be relevant for being selected. In male athletes, the binomial logistic regression model was also statistically significant (*χ*² (3) = 11.38, *p* = .010) with explaining 14.2% (Nagelkerke's *R*^2^) of the variance in selection status but only the anthropometric- and maturation-related component (*p* = .004) predict selection.

**Discussion:**

Anthropometric conditions (such as body height, body weight, and wingspan) and the maturity status are particularly important in talent selection in 3 × 3 basketball for both sexes. Regarding motor-performance variables, we found a predictive value for talent selection only in females (without sprinting), but not in males which means that more ‘athletic' female athletes seem to be favoured in talent selection. The results suggest that the talent selection process might be biased by maturation status even in middle adolescence. Therefore, coaches who decide on athletes’ selection should be aware of the temporal advantages induced by earlier maturation when evaluating talented athletes and should consider strategies such as bio-banding to evaluate the real and potential value of talented athletes.

## 1 Introduction

In many team sports such as volleyball, handball, or basketball, there is an increasing interest in new ways of implementation apart from the classic ones. For example, in basketball, there is a variation called streetball that is played three vs. three on a half court with only one basket. It is characterized by an intermittent profile with a 1:1 work: rest ratio, with games lasting approximately 15 min and short ball possessions (6–8 s). Better shooting and defensive efficiency or low number of turnovers are some of the key technical-tactical indicators of the performance profile in a physically demanding game with quick actions such as accelerations, decelerations, changes of direction and explosive jumps [for an overview (1)]. In the late 2000s, the International Basketball Federation (FIBA) started its first attempt to institutionalize streetball or so-called “3 × 3 basketball” with standardized rules. Subsequently, the game experienced a considerable increase in popularity and global participation with 182 national federations becoming involved, finally leading to its inclusion in the Tokyo Olympic games in 2021 (2).

Hence, it is not surprising that 3 × 3 basketball is becoming more and more professionalized with increasing interest in talent development aiming to compete and win at the highest (international) level. Typically, one of the first steps in this talent development process is to select the most talented athletes in terms of sport-specific requirements such as motor performance or anthropometric conditions (3, 4). Talent selection refers to the on-going process of choosing athletes within the development program who meet relevant sport-specific requirements to progress into a future team (3). Whereas there are currently no studies in 3 × 3 basketball that have investigated relevant factors in the talent selection process, there are already many analyses in “classic” basketball (5v5) that have identified different factors with a relevance for the selection of young basketball athletes (5–8). One of these factors are anthropometric conditions such as body height, body weight, or wingspan (8, 9). For example, Soares et al. (10) showed that among young female basketball athletes (between 10.5 and 15.5 years), taller athletes are selected more often for higher competitive levels. Also, Torres-Unda et al. (8) investigated 13- to 14-year-old male basketball athletes and found that elite athletes were taller compared to nonelite athletes. In general, the importance of height and other body dimensions (body weight, wingspan) are well documented in basketball (11, 12). Looking at senior basketball, some studies have shown that body height is one of the main predictors of performance (11), and teams with taller athletes are ranked higher in the FIBA World Cup compared to teams with shorter athletes (13). Such performance predictors are closely linked to coaches’ decisions to pick players for the highest level. For example, Cui et al. (14) show that body height is an essential prerequisite for being picked in the National Basketball Association (NBA) draft.

Besides anthropometric factors, other important aspects in talent selection are the physical and physiological capacities (15). Basketball is characterized by different strength- and power-related actions such as jumping, sprinting, or shuffling (16, 17). Therefore, motor performance seems to be essential for elite (youth) athletes. Torres-Unda et al. (8) underlined the importance of physical fitness and found that selected athletes outperform non-selected athletes in physical tests such as sprints, jumps, and endurance tests. Joseph et al. (18) postulated the relevance of power-related motor-performance variables and demonstrated that selected female athletes in certain age cohorts show better performance in 20 m sprint time or countermovement jump (CMJ) height than non-selected athletes. Also, Ramos et al. (7) showed that athletes in higher-ranked teams perform better in agility performance tests than those in lower-ranked teams. 3 × 3 basketball is also characterized by a fast pace with numerous quick and high-intensity actions. However, medium- to high-intensity actions during live time are greater in 3 × 3 basketball than in 5v5 basketball [for an overview (1)]. More specifically, 3 × 3 athletes perform more change of directions, accelerations, and decelerations per minute (19). Furthermore, competitions are organized in tournaments with several games per day on consecutive days (20), and this imposes a high physical load. Therefore, the importance of motor performance could be even greater in 3 × 3 basketball than in 5v5 basketball.

In addition to these two factors, Malina et al. (21) postulated that the talent selection process is biased by biological maturation–that is, the progress toward the adult or mature state. The biological maturity status of individuals with the same chronological age can vary widely, especially during different stages of adolescence (between 10 and 16 years). These differences depend on various factors such as the time of fastest upward growth defined as the age of peak height velocity (APHV). Whereas females reach their APHV at approximately 12 years, males reach it at around 14 years (22). Nevertheless, this can vary from individual to individual, with some reaching this growth spurt several years later or earlier than others (23). APHV is related to major changes in different systems. For example, previous studies have shown that growth and sex hormones strongly increase in concentration at this stage (24, 25), and that they influence anthropometric conditions (e.g., body height, body weight, body segments) and/or motor-performance variables [e.g., strength (26)]. This can result in large differences between children and young adolescents of the same chronological age in terms of anthropometric conditions and motor-performance variables (27). For the process of talent selection, these interindividual differences between adolescents can lead to temporary advantages or disadvantages in their age cohorts (28, 29). Early-matured athletes (meaning APHV is low) could be taller and stronger and therefore will be selected more often than late-matured athletes (meaning APHV is high) (5, 30).

In recent years, more and more studies have investigated the influence of maturity status on different anthropometric and motor-performance-related variables as well as on talent selection in team sports (5, 31–34). Most studies found that—expectedly—early-matured athletes performed better in motor-performance tests, were taller, and were selected more often for squads [for an overview (27)]. In basketball, some studies investigated the influence of maturity status and found advantages for early-matured adolescents on anthropometric and motor-performance variables, as well as in career progression. For example, Arede et al. (31) demonstrated that basketball athletes with a lower APHV (early-matured) are taller and heavier than athletes with a higher APHV (late-matured). Guimarães et al. (35) showed advantages in sprint or change-of-direction (COD) test as well as in anthropometric conditions (e.g., body height, body weight) for early maturation groups. Also, according to Ribeiro Junior et al. (9), early-matured athletes had a greater chance of reaching the highest national league. However, these maturation-related benefits are temporary and diminish as the athletes mature. For example, early-matured females and males do not exhibit a higher final body height than their on-time or late-matured peers (36, 37). In addition, Lefevre et al. (38) found that the late-matured males not only caught up with the early-matured males in adulthood, but also performed better in explosive strength tests.

Most of these studies examined the maturity status in male athletes in early stages of adolescence (between 12 and 14 years)–that is, at the time when the growth spurt often sets in. But there are also some studies on the influence of maturation after the growth spurt in middle or late adolescence (15−16 years). These could still find advantages for groups with a low APHV. For example, Luna-Villouta et al. (39) and Vandendriessche et al. (40) found that interindividual differences related to biological maturation are still present at the ages of 15−16 years. In this context, Arede et al. (5) reported on the influence of maturity status in 5v5 basketball at an age of around 15 years and concluded that maturity is a decisive factor in the selection of the “under 16” national team. Consequently, maturity status could influence talent selection processes at all stages of adolescence and needs to be considered to ensure a fair system where talented athletes are prevented from not being selected due to later maturation (41). This is particularly important when considering effects such as those described in the “underdog hypothesis”, which proposes that later maturing athletes need to be more advanced in technical-tactical skills to remain competitive in talent selection processes (42). However, earlier maturation could result in the selection of less skilled athletes to the detriment of more skilled but later maturing athletes. Kelly et al. (43) also found support for such effects in 5v5 basketball. Nevertheless, in recent years, the FIBA has organized international 3 × 3 basketball youth tournaments for certain age groups. Therefore, in Germany, talent development and selection in 3 × 3 basketball are still organized in annual age groups with specific cut-off dates based on an athlete's chronological age. Although the approach of classifying teams into certain age groups (e.g., “under 17” meaning the athletes are at the age of 16 years or younger) is intended to provide a fair system of practice and competition (44), it does not take biological maturation into account and therefore does not ensure fair conditions for all.

To sum up, talent selection is based on a wide range of factors, but anthropometric conditions, motor-performance variables, and maturity status seem to be the relevant factors in “classic” basketball. In 3 × 3 basketball, to the best of our knowledge, there is still no research investigating which factors in the talent selection process are mainly used to distinguish between selected and non-selected athletes (for females and males). Therefore, the aim of this study was to investigate the main predictors of selection and non-selection in male and female athletes. Based on current literature in 3 × 3 (19, 20) as well as on studies in classic 5v5 basketball (5, 7, 8), we hypothesized that anthropometric conditions and motor-performance variables would play a crucial role in the talent selection process and explain the variance in selected and non-selected athletes. Furthermore, even though talent selection in 3 × 3 basketball starts very late (beginning at “under 17” national team) compared to other team sports, we hypothesized that maturation status would have a major influence on selection.

## 2 Material and methods

Data were collected in September 2021 and September 2022 in two separate cohorts of athletes. All measurements were carried out as a part of the 3 × 3 basketball “under 17” national selection tournament of the German Basketball Federation (Deutscher Basketball Bund, DBB). This annual tournament is one part of a multi-stage selection process with the aim to form the 3 × 3 basketball “under” 17 youth national team. It is the very first stage of talent selection at the national level. In order to be invited to this event, all athletes must be part of their respective 3 × 3 basketball state teams (e.g., Bavaria, Lower Saxony), for which they are selected by qualified regional coaches mainly on the basis of their performance in competitions in 5v5 basketball as well as in 3 × 3 basketball. This means this population is pre-selected at regional level and sent to national selection tournament by the state federations. Approximately one third of the athletes participating in the annual national tournament are selected based on coaches’ evaluations of the athletes’ performance at the national selection tournament. These athletes will then be invited to the so-called “selection training program”, where the final 3 × 3 basketball “under 17” youth national team is formed.

Each nation can send one national team to the international championships, usually consisting of four players in 3 × 3.

### 2.1 Participants

A total of 192 German young female and male athletes (*M*_age_ = 16.11 ± 0.45 years; *n* = 85 were female) were included in this study. All athletes were in reportedly good physical condition. Three participants were partly excluded (two female athletes and one male athlete) because they could then not perform the whole test battery due to an existing injury. One female did only perform the chest-pass test and the other female did not perform the change-of-direction (COD) test. The male athlete could not perform the drop-jump (DJ) test. These athletes were included in the analysis for the tests in which they participated. All athletes were part of 3 × 3 basketball “under 17” youth state teams and participated in a developmental program of their respective state team. However, all athletes started to compete in 5v5 basketball and still compete in it, but the developmental programs and youth teams (state and national) are separate. Athletes and their parents or legal representative(s) were informed based on the declaration of Helsinki about the testing protocols and gave written consent. The procedures were approved by Goethe University Frankfurt am Main, Department of Psychology and Sports Science, Ethics Committee (2021-30, June 28, 2021).

### 2.2 Procedure

Upon arriving and after being informed about the schedule, athletes’ birth dates were collected. Subsequently, athletes were assessed in anthropometry following a standardized physical warm-up and motor-performance tests. All tests were performed in an indoor sports facility.

### 2.3 Measures

#### 2.3.1 Anthropometry

Similar to previous investigations in 5v5 basketball (6, 32, 45), anthropometric assessments consisted of measurements of body height in cm, sitting height in cm, leg length in cm, wingspan in cm, and body weight in kg. Body height was measured using a laser rangefinder (±0.1 cm; Bosch, Gerlingen, Germany); sitting height and leg length, using a measuring tape (±0.1 cm; Busduga, Leutkirchen, Germany); and wingspan, using a measuring tape (±0.1 cm; Stanley, Idstein, Germany). Body weight was measured in sports clothing without shoes and was assessed using a digital scale (±0.1 kg; Bohmann, Hamburg, Germany). Following Mirwald et al.'s (46) procedure, maturity status (APHV) was calculated with the BioFinal 3.4 software (47) that uses sex-specific formulae. APHV was used for further analyses.

#### 2.3.2 Sprint test

Athletes performed two maximal sprints of 20 m to assess sprint performance (48). Split time at 5 m and 10 m as well as at 20 m were measured with a photocell system **(**Microgate, Bolzano, Italy**)**. Athletes were instructed to carry out a self-initiated start from a standardized standing position one meter behind the starting line. They performed two trials of sprints. If the two trials were too far apart from each other (>10%), they performed a third trial. Between trials, athletes had a short break (1.5 min). The fastest time trial at 5 m split time was used for further analyses. Analyses were based on the time for 5 m, the time between 5 m and 10 m, and the time between 10 m and 20 m.

#### 2.3.3 Vertical jump-and-reach test

Maximal vertical jump-and-reach height was assessed with a self-constructed device similar to the device used by Muehlbauer et al. (49). The height of the lowest plastic swivel vane was measured with a laser rangefinder (Bosch, Gerlingen, Germany). The plastic swivel vanes had a thickness of 1.0 cm. Athletes were instructed to jump from the side of their dominant hand (lefthanders start from the left side; righthanders start from the right side). Jumps were performed inside a 3 m area without any other instructions except for jumping as high as they could and reaching the highest plastic swivel possible. The athletes performed two jumps. A third jump was performed when the first two were more than 10% apart from each other. The highest jump was used for further analyses.

#### 2.3.4 Countermovement jump (CMJ)

Maximal CMJ height was assessed using a photocell system [(50); OptoGait, Micogate, Bolzano, Italy]. Athletes performed two maximal CMJs. Jumps were standardized with hands on the hips, a knee flexion angle of about 90°, and the instruction to jump as high and explosively as possible. The highest jump was used for further analyses.

#### 2.3.5 Drop jump (DJ)

Maximal DJ height and contact time were assessed to calculate the reactive-strength index (RSI) according to the protocol of Markwick et al. (51) and using a photocell system (OptoGait, Micogate, Bolzano, Italy). Athletes performed two bilateral DJs from a 24 cm high box. All jumps were performed in a standardized fashion with the hands on the hips. Athletes were instructed to lean forward with one leg (leg could be chosen by athletes) and let themselves drop to the ground. As soon as they hit the ground, they should jump as quickly as possible and as high as possible. The jump with the highest RSI was used for further analyses.

#### 2.3.6 Change-of-direction (COD) test

To test the ability to change directions, athletes performed a COD test by using a photocell system (±0.1 s; WK, Ditzingen, Germany). Setting and test protocol was based on Willberg et al. (52). Athletes performed the COD test twice. If the deviation between both test trials was too high (>10%), a third test trial was carried out. The fastest run was used for further analyses.

#### 2.3.7 Chest-pass test

Chest-pass test was used in similar studies to measure the explosive upper-body strength (7, 53). The chest-pass test was carried out on a basketball court with an official 3 × 3 basketball (Wilson Sporting Goods, Chicago, United States of America). Athletes stood in a standardized position behind the line. The instruction was to hold the ball at chest level and throw it as far as possible with both hands in a linear way. The length was recorded with a measuring tape (±0.1 m; Stanley, Idstein, Germany). The best result was used for further analysis.

### 2.4 Statistical analysis

Data were analyzed with IBM SPSS statistics Version 29.0.0.0 for Macintosh (IBM, New York, USA). All analyses were computed for each sex separately.

To avoid overestimation of the binomial logistic regression model, data were reduced by using principal component analysis (PCA) with varimax rotation. We used varimax rotation to simplify the factors by maximizing the variance of loadings within factors, across variables, for easier interpretation of the components. Afterwards, the resulting components of the PCA were analyzed in a binomial logistic regression to determine which components predicted talent selection (54). The selection status of an athlete was coded in binary form and used as the dependent variable. The descriptive data are presented as means and standard deviations. The significance level was set *a priori* at *p* < .05.

## 3 Results

Descriptive data from all tests for female (*n* = 85) and male athletes (*n* = 107) are shown in Table 1.

**Table 1:** Descriptive data from all sociodemographic, anthropometric, and motor-performance variables for both sexes.

		Female				Male		
		Selected		Non-selected		Selected		Non-selected
Variable	*n*	M ± SD	*n*	M ± SD	*n*	M ± SD	*n*	M ± SD
Chronological age	30	15.99 ± 0.52	55	16.07 ± 0.43	34	16.12 ± 0.47	73	16.19 ± 0.41
Age of peak height velocity	30	12.26 ± 0.49	55	12.48 ± 0.44	34	13.18 ± 0.76	73	13.50 ± 0.64
Birth quartile (1st/2nd/3rd/4th)	30	23%/13%/43%/20%	55	27%/27%/29%/16%	34	35%/26%/15%/24%	73	42%/19%/22%/16%
Body height (cm)	30	176.83 ± 7.60	55	173.85 ± 6.69	34	190.43 ± 7.84	73	185.87 ± 7.79
Body weight (kg)	30	70.51 ± 9.34	55	67.40 ± 9.78	34	81.39 ± 10.83	73	76.36 ± 10.89
Wingspan (cm)	30	181.01 ± 8.93	55	176.79 ± 8.12	34	197.34 ± 7.65	73	191.97 ± 9.30
Chest pass (cm)	30	11.21 ± 1.22	55	10.53 ± 0.90	34	14.73 ± 1.61	73	14.21 ± 1.61
Jump and reach (cm)	30	53.92 ± 5.56	54	49.75 ± 5.38	34	73.70 ± 8.41	73	70.37 ± 6.44
CMJ (cm)	30	31.40 ± 4.85	54	27.81 ± 4.12	34	37.69 ± 5.73	73	37.22 ± 5.14
DJ RSI	30	1.39 ± 0.32	54	1.21 ± 0.29	34	1.55 ± 0.37	72	1.51 ± 0.34
Sprint 0–5 m (s)	30	1.06 ± 0.11	54	1.06 ± 0.08	34	0.98 ± 0.08	73	0.99 ± 0.08
Sprint 5–10 m (s)	30	0.80 ± 0.03	54	0.82 ± 0.04	34	0.74 ± 0.03	73	0.75 ± 0.04
Sprint 10–20 m (s)	30	1.43 ± 0.07	54	1.48 ± 0.06	34	1.31 ± 0.06	73	1.32 ± 0.06
COD test (s)	30	7.90 ± 0.53	53	8.10 ± 0.47	34	7.28 ± 0.39	73	7.31 ± 0.44

CMJ, countermovement jump; DJ RSI, drop jump reactive strength index; COD, change of direction.

### 3.1 Predictors of talent selection

#### 3.1.1 Principal component analysis (PCA)

A PCA (varimax rotation) was calculated to reduce the number of variables for the following binomial logistic regression in order not to overload the model. The Kaiser-Meyer-Olkin test of sampling adequacy (KMO) and the Bartlett test of sphericity were calculated for each sex to check whether the data were suitable for PCA. According to Hair et al. (55), practical significance is given when the component loading is ≥ ± .500. Therefore, only component loadings ≥ ± .500 were linked to a component.

For female athletes, KMO was.75 and the Bartlett test of sphericity was *p* < .001, *χ*² (66) = 565.341. Concerning the Kaiser rule, PCA revealed three components with an eigenvalue greater than 1.0 (55). Additionally, a scree plot (56) favoured a three-component solution. This three-component solution explained a cumulative variance of 69.43% (1st component: 35.97%; 2nd component: 23.52%; 3rd component: 9.93%). Chest pass, vertical jump and reach, CMJ, sprint 5–10 m, sprint 10–20 m, DJ RSI, and the COD test loaded on the first component (general motor-performance component); age of peak height velocity (APHV), body height, body weight, and wingspan, on the second component (anthropometric- and maturation-related component); and sprint 0–5 m, sprint 5–10 m, and sprint 10–20 m, on the third component (sprint component). Component loadings for female athletes are shown in Table 2.

**Table 2:** Principal component loadings for female athletes.

Item		Component	
	1	2	3
APHV		−.842	
Body height		.906	
Body weight		.768	
Wingspan		.905	
Chest pass	.519		
Jump and reach	.810		
CMJ	.817		
Sprint 0–5 m			.846
Sprint 5–10 m	−.455		.660
Sprint 10–20 m	−.554		.678
DJ RSI	.773		
COD test	−.731		

Rotation method: varimax with Kaiser normalization. Loadings smaller than 400 are not represented. APHV, age of peak height velocity; CMJ, countermovement jump; DJ RSI, drop jump reactive strength index; COD, change of direction.

For male athletes, KMO was.71 and the Bartelett test of sphericity was *p* < .001, *χ*² (66) = 688.92. Similar to the PCA of female athletes’ data, three components with an eigenvalue greater than 1.0 were found and the scree plot also favoured a three-component solution. This three-component solution explained a cumulative variance of 68.18% (1st component: 32.44%; 2nd component: 26.47%; 3rd component: 9.28%). APHV, body weight, body height, and wingspan loaded on the first component (anthropometric- and maturation-related component); vertical jump and reach, CMJ, sprint 5–10 m, sprint 10–20 m, DJ RSI, and the COD test, on the second component (general motor-performance component); and chest pass, sprint 0–5 m, and the COD test, on the third component (muscular-coordination component). Component loadings for male athletes are shown in Table 3.

**Table 3:** Principal component loadings for male athletes.

Item		Component	
	1	2	3
APHV	−.858		
Body height	.899		
Body weight	.843		
Wingspan	.891		
Chest pass			−.776
Jump and reach		.855	
CMJ		.881	
Sprint 0–5 m			.706
Sprint 5–10 m		−.701	
Sprint 10–20 m		−.743	
DJ RSI		.623	
COD test		−.402	.561

Rotation method: varimax with Kaiser normalization. Loadings smaller than 400 are not represented. APHV, age of peak height velocity; CMJ, countermovement jump; DJ RSI, drop jump reactive strength index; COD, change of direction.

#### 3.1.2 Binominal logistic regression

The results of the binomial logistic regression are summarized in Table 4 for both sexes. Multicollinearity was checked according to Tabachnick and Fidell (54) but was not present (*r* > .90) for either sex. For female athletes, the overall model was statistically significant, *χ*² (3) = 26.86, *p *< .001, and included the general motor-performance component (*p *< .001) and the anthropometric- and maturation-related component (*p *= .004). The model explained 37.9% (Nagelkerke's *R*²) of the variance in selection status. It correctly classified 73.5% of the female athletes and the success rate of predicting non-selected athletes was 83.0% while it was 56.7% for selected athletes. For male athletes, the overall model was also statistically significant, *χ*² (3) = 11.38, *p *= .010, but included only the anthropometric- and maturation-related component (*p *= .004). The model explained 14.2% of the variance (Nagelkerke's *R*²) in selection status, and it correctly classified 69.8% of the male athletes. The success rate for predicting non-selected athletes was 90.3% and for selected athletes 26.5%.

**Table 4:** Binomial logistic regression for female and male athletes*.*

Sex	Variable	B (*SE*)	Wald	*df*	*p*
Female	Constant	0.825 (.290)	8.079	1	.004
	General motor-performance component	−1.189 (.341)	12.168	1	<.001
	Anthropometric- and maturation-related component	−0.903 (.317)	8.108	1	.004
	Sprint component	0.491 (.280)	3.081	1	.079
Male	Constant	0.861 (.230)	14.044	1	<.001
	Anthropometric- and maturation-related component	−0.737 (.253)	8.477	1	.004
	General motor-performance component	−0.266 (.220)	1.458	1	.227
	Muscular-coordination component	0.163 (.218)	0.559	1	.455

## 4 Discussion

The aim of the present study was to identify the main predictors in the talent selection process for 3 × 3 basketball that distinguish between selected and non-selected athletes. We hypothesized that anthropometrics, motor-performance variables, and maturity status would explain the variance in the talent selection process. As expected, our results show that anthropometric conditions (such as body height, body weight, and wingspan) and the maturity status seem to be relevant factors for selection in both sexes. This means that selected female and male athletes were taller, heavier, had a greater wingspan, and an advanced maturity status compared to non-selected athletes. Regarding the motor-performance variables, we found a predictive value for talent selection only in female athletes (without sprinting), but not in male athletes.

Results on anthropometric conditions are in line with the current literature on classic basketball that also shows advantages for selected athletes in different anthropometric conditions. For example, selected athletes are taller (6), heavier (57), and have a greater wingspan (12) than non-selected or lower-ranked athletes. Many studies (6, 9, 35, 58) have investigated classic youth basketball athletes in different age groups and indicated the importance of being tall in the talent selection process in all age groups (ranging from 10 years to 16 years). Ribeiro Junior et al. (9) examined the career progression of youth athletes and found that being tall is one of the main reasons for reaching the highest league. Also, Cui et al. (14) examined the draft of the NBA and postulated that even in the transition to senior basketball, being tall is a key determinant for being drafted. Consequently, anthropometric conditions seem to be very relevant in basketball and are also related to game performance and success. In this context, some studies have examined the relationship between technical actions and found that taller athletes have advantages in different technical skills such as rebounding, passing, or shooting compared to their shorter peers (8, 11, 59). For example, Garcia-Gil et al. (11) found that technical actions such as shooting or passing correlate significantly with greater wingspan. These anthropometric characteristics are important in basketball for both defensive actions (e.g., covering wider and higher space or blocking shots from shorter athletes) as well as offensive actions (e.g., shooting or dunking). Comparing 3 × 3 with 5v5 basketball, the number of passes, shots, and rebounds in 3 × 3 basketball is higher compared to the classic variation (60). Due to an increased frequency of technical actions as well as a reduced number of players, the importance of anthropometrics could be even greater in 3 × 3 compared to 5v5 basketball.

Based on this, it is not surprising on the one side that coaches focus on height and other anthropometric conditions in the talent selection process. But, on the other side, the results of this study and of previous research (5, 7, 8) show that anthropometric conditions correlate with maturity status. This means that early-matured athletes are taller, heavier, and have a greater wingspan than late-matured athletes. This is shown especially for early adolescence (around growth spurt), but our results demonstrate an influence even in middle adolescence. The talent selection process in 3 × 3 basketball is very late compared to, for example, football (starting around two to three years earlier). The chronological age of the athletes in this study was 16.04 (females) and 16.16 (males), which means that they were in middle adolescence and past their APHV, which was at age 12.40 in females and at age 13.40 in males. Consequently, the talent selection process could be biased by maturation status in all stages of adolescence. In 5v5 basketball, different studies have already shown that early-matured athletes tend to be selected in preference to late-matured athletes (5, 8, 57). In this context, Leyhr et al. (6) examined the influence of different factors of the talent selection process in 5v5 basketball in Germany and found significant differences in selection status with advantages for those athletes who had a lower APHV. This was also shown in a study by Arede et al. (5), who examined the discriminating variables in the Portuguese “under 16” national team. They postulated that maturity status seems to be a key variable in 5v5 basketball, and that it increases the probability of selection. Torres-Unda et al. (59) demonstrated that early-matured athletes dominate in higher-performing 5v5 basketball teams (finalists and semifinalists) compared to lower-performing teams (quarter finalists). Overall, our results are in line with previous studies suggesting a bias even in middle or late adolescence. In addition, it should be noted that the equation formula of Mirwald et al. (46) for estimating the APHV tends to become more inaccurate with increasing chronological age (61). For instance, Kozieł and Malina (62) showed that predicted APHV is systematically later than observed APHV in early-matured athletes and systematically earlier than observed APHV in late-matured athletes. This may also explain why our estimates of APHV for male athletes were slightly higher and our estimates for female athletes were considerably higher than the average APHV in the population of athletes of different sports [for an overview, see (63, 64)]. Therefore, the maturity bias could be even greater. However, knowing the limitations of Mirwald's (46) formula, it is still a time, cost-effective and non-invasive method that does not require any measurements from people other than the athlete (e.g., parental height), as it is the case with Khamis and Roche's equation (65), and does not require any special or costly equipment, as it would be needed to determine skeletal age (23).

Even though maturity status positively influences anthropometric conditions temporarily, different studies show no advantages in terms of anthropometric conditions for early-matured female or male athletes when they are fully mature. Vizmanos et al. (36) postulated that maturity status does not affect final height in females or males. In comparison, Chen et al. (37) even found that late-matured females and males tend to have higher body height at 18 years than early-matured females and males. Nonetheless, results of both studies highlight the importance for coaches and scouts to be aware of the effects of biological maturation and refrain from overestimating (temporally) height differences. Considering that in senior 5v5 basketball, taller athletes still perform superiorly regardless of position (13), it is crucial to avoid deselection of young athletes because of temporary height differences. Furthermore, considering that Kelly et al. (43) found “underdog” effects in 5v5 basketball, late-matured athletes may reveal their more competitive technical-tactical skills as temporary height differences fade. Considering that our results show that the more mature, and therefore taller, athletes are favoured in the selection process, it is particularly important to make coaches aware of temporal height differences as well as “underdog” effects. Otherwise, coaches might deselect late-matured athletes without recognizing their true potential. In this context, Malina et al. (66) showed that when athletes were grouped by their maturity status (bio-banding) and temporal advantages disappeared, coaches noted aspects of play not ordinarily seen due to their dependence on size.

Contrary to our expectations, we found inconsistent results for the motor-performance-related component. Whereas the general motor-performance component is also included in the model (37.9% of variance; without sprints) for female athletes, it is surprising that no motor-performance-related component was included in the male model. Most studies on associations between motor performance and success in talent selection processes found a positive influence of motor-performance variables. In this context, Torres-Unda et al. (8) examined male youth athletes and highlighted the relevance of speed-related outcomes for predicting future success in 5v5 basketball. Also, Ramos et al. (7) found that male and female athletes from higher-performing teams performed better in different motor-performance tests (e.g., sprint, COD test, upper-body power test) than athletes from lower-performing teams. This underlines the relevance of power-related performance. Especially in 3 × 3 basketball, we would expect these variables to be relevant, because 3 × 3 athletes perform even more CODs, accelerations, and decelerations per minute compared to 5v5 athletes (1, 19). But in contrast to this literature and our expectations, our findings in male athletes reveal no predictive value of the motor-performance variables for talent selection. One possible explanation could be linked to the system of talent selection process in Germany, which is organized in a two-step selection procedure. In the first step, all associations of the respective states select talents from their regional team. Then, the second step consists of a final selection for the national team. Most studies that found an influence of motor-performance variables investigated the first-step selection. In this study, only the second step of the selection process was examined, meaning all invited athletes had been preselected. We speculate that there are motor-performance benchmarks that an athlete must reach to successfully pass the first-step selection. This is why this group of athletes becomes more homogeneous in the second-step selection, indicating that the importance of the motor-performance components may become more and more negligible. In this context, Leyhr et al. (6) examined 5v5 preselected basketball athletes who played in a selection tournament for the “under 15” German national team and also found similar results. In addition, they elucidated no differences in sprinting or COD performance between selected and non-selected male athletes, but differences in jumping performance.

However, results for female athletes are in line with the current literature, and motor-performance tests differentiate between selected and non-selected athletes even in the second-step selection. One possible explanation could be the number of active participants or the depth of the competition (e.g., the more players competing for finite number of starter places in a single team, the more likely that at earlier stages of talent selection motor-performance benchmarks are reached). The number of male athletes is considerably greater than that of female athletes (*n*_males_ = 107, *n*_females_ = 85), meaning that the motor-performance benchmarks for female athletes are reached in different steps of selection. Similar tendencies can be seen in other domains such as the relative age effect. In a systematic review, de la Rubia et al. (67) postulated that in women's sports, even with an overrepresentation of relatively older athletes, relative age did not have an influence on competition performance due to factors such as the number of active participants or the depth of the competition (e.g., reduced number of female athletes competing for a limited number of places on a team compared to male athletes).

A further explanation for different findings in male and female athletes could be that the general motor-performance component for males is not as important in the talent selection process as for female athletes because of factors that could be linked to technical aspects of the game. As mentioned before, technical actions such as shooting correlate with greater wingspan, but also with upper-body strength (11, 53). Because female athletes have smaller wingspan than male athletes, this could be a reason why chest-pass performance, which is part of the general motor-performance component and correlates with upper-body strength (68), is more important in female athletes than in male athletes. Furthermore, due to the increased number of shots in 3 × 3 basketball, the number of rebounds in 3 × 3 basketball is higher too (60). Because rebounds are considered to be a key performance indicator for predicting the outcome of the game (69), it is advantageous to be tall (11) and/or to jump high (70) to get these rebounds. Again, female athletes are smaller than male athletes, so jumping performance, which is a main part of the general motor-performance component, could have more relevance for them.

### 4.1 Limitations

To avoid overfitting the model, we included principal components from the PCA in the binomial logistic regression rather than using all variables in isolation. This allows to consider and include as much data as possible in an overall analysis. However, it does not allow conclusions about the exact contribution of specific variables.

The overall model of the binomial logistic regression statistically discriminated between the selection status of female and male athletes. Nevertheless, the success rate for predicting selected athletes was 56.7% for female athletes and only 26.5% for male athletes. This suggests that other factors such as coaches’ assessments [often referred to as coach's eye (71)], tactical aspects [e.g., positioning (32)], technical skills (e.g., shooting qualities), or psychological factors (e.g., decision-making) might also play an important role for game performance. For homogeneous populations who have passed their APHV a longer time ago, these factors could have a greater impact on selection decisions (18, 72, 73) because all players have already reached a high degree of their maturational development. Thus, these variables should be considered in future investigations.

## 5 Conclusion

The aim of this study was to analyze predictors for selection in a 3 × 3 talent recruitment setting. For female athletes, the main predictors in 3 × 3 basketball talent selection are the anthropometric- and maturation-related and the general motor-performance components. For male athletes, in contrast, only the anthropometric- and maturation-related component seems to be relevant. Furthermore, early-matured female and male athletes show advanced anthropometric conditions compared to late-matured athletes. In this context, Soares et al. (10) assume that coaches probably tend to overvalue body height when selecting youth athletes. Therefore, coaches should raise their awareness to avoid deselecting late-matured athletes just because they have a temporal disadvantage in terms of their anthropometric conditions. Strategies such as bio-banding, in which athletes are matched with equivalent opponents in terms of maturity level (66) should be considered to retain late-maturing athletes in a competitive system. Arede et al. (74) suggest that in 5v5 basketball with bio-banded teams, the real and potential value of talented youth athletes could be better understood, and hence the accuracy of talent selection processes could be improved.

## Data Availability

The raw data supporting the conclusions of this article will be made available by the authors, without undue reservation.
